# Inhibition of the CRF_1_ receptor influences the activity of antidepressant drugs in the forced swim test in rats

**DOI:** 10.1007/s00210-017-1377-0

**Published:** 2017-04-20

**Authors:** Andrzej Wróbel, Anna Serefko, Aleksandra Szopa, Karol Rojek, Ewa Poleszak, Krystyna Skalicka-Woźniak, Jarosław Dudka

**Affiliations:** 1 Second Department of Gynecology, Medical University of Lublin, Jaczewskiego 8, 20-090 Lublin, Poland; 2 Chair and Department of Applied Pharmacy, Medical University of Lublin, Chodźki 1, 20-093 Lublin, Poland; 3 Department of Pharmacognosy with Medicinal Plants Unit, Medical University of Lublin, Chodźki 1, 20-093 Lublin, Poland; 4 Department of Toxicology, Medical University of Lublin, Jaczewskiego 8B, 20-093 Lublin, Poland

**Keywords:** CRF_1_ receptor antagonist, Imipramine, Fluoxetine, Forced swim test, Corticotropin-releasing factor level, Rats

## Abstract

Hyperactivity of the hypothalamic-pituitary-adrenal axis (HPA) and impairment of the central corticotropin-releasing factor (CRF) system are factors in the pathogenesis of depression. Though several antagonists of the CRF_1_ receptor were effective in the recognized behavioral tests for antidepressant activity, there is still little information on the potential interactions between CRF_1_ receptor inhibitors and conventional antidepressant therapy. The aim of our study was to assess the influence of SN003, a CRF_1_ receptor blocker, on the activity of imipramine and fluoxetine in the forced swim test (FST) in rats which presented some signs of depression. The experiments were carried out on female Wistar rats subjected to 14-day subcutaneous corticosterone (CORT) administration (20 mg/kg/day). The antidepressant-like effect was determined by the FST and the CRF levels in the hypothalamus, amygdala, and peripheral blood were measured by a high-sensitivity immunoenzymatic test. SN003 (0.5 mg/kg) potentiated the antidepressant-like effect of imipramine (15 mg/kg) and fluoxetine (7.5 mg/kg). Moreover, the co-administration of the tested agents abolished CORT-induced increase in CRF levels in the examined biological material more profoundly than monotherapy. Our present findings give further evidence that the blockage of CRF action may be useful in the treatment of mood disorders. The concurrent use of well-known antidepressants with CRF_1_ receptor antagonists could be beneficial in terms of safety, since it requires lower doses of the applied agents.

## Introduction

Literature data indicate that stressful life events may predispose to the development of depression. Hyperactivity of the hypothalamic-pituitary-adrenal (HPA) axis (the stress axis) has been observed in depressed patients, and it is regarded as an important factor in the pathogenesis of this disease. While HPA axis is crucial for the endocrine response to a stressor, the corticotropin-releasing factor (CRF), also known as the corticotropin-releasing hormone, plays an important role in regulating this neuroendocrine reaction. CRF produced in the parvocellular neurons of the paraventricular nucleus in the hypothalamus serves as a hormone, whereas CRF produced in other brain regions serves as a neurotransmitter (Chappell et al. 1986). In a stressful situation, increased synthesis and release of CRF is observed, which in turn triggers the release of the adrenocorticotropic hormone (ACTH), followed by the higher synthesis and release of glucocorticoids—cortisol (in primates) or corticosterone (in rodents). In healthy subjects, glucocorticoids send a negative feedback which causes a decrease in the synthesis and release of CRF, ACTH, and cortisol/corticosterone. In depressed subjects, the HPA negative feedback loop may be impaired (Galard et al. 2002). Elevated CRF levels result not only in neuroendocrine changes but also in behavioral and physiological changes similar to those recognized as a response to stress (i.e., increased heart rate, sleeplessness, suppression of exploratory behavior in an unfamiliar environment, grooming behavior, decreases in sexual interest, and food intake) (Heinrichs and Koob 2004).

CRF acts through two different CRF receptors (CRF_1_ and CFR_2_) which are involved in the modulation of anxiety- and depression-related behavior (Takahashi et al. 2001). Particular attention was given to CRF_1_ receptors as targets for substances with potential antidepressant activity. A high CRF1 receptor density has been detected in the cerebral cortex, cerebellum, olfactory bulb, medial septum, hippocampus, amygdala, and pituitary (Gilligan et al. 2000). Several authors have demonstrated that CRF_1_ receptor antagonists were effective in recognized behavioral tests (i.e., the rat forced swim test, FST; the tail suspension test, TST) and animal models (i.e., the learned helplessness paradigm, the olfactory bulbectomy model, the chronic mild stress model, the chronic adolescent stress) evaluating the antidepressant-like effect of novel agents (Bourke et al. 2014; Chaki et al. 2004; Griebel et al. 2002; Mansbach et al. 1997). In our previous studies, we also demonstrated that a high-affinity non-peptidic CRF_1_ receptor blocker which displays >1000-fold selectivity over CRF_2_ receptors—SN003—possesses antidepressant-like activity comparable to that obtained with typical antidepressant drugs (Wrobel et al. 2016). Non-peptide compounds seem to be of particular value, as the penetration of the peptide-based CRF receptor antagonists through the blood-brain barrier is not sufficient (Mansbach et al. 1997). Though most of the clinical trials on the antidepressant efficacy of CRF_1_ receptor blockers were discontinued because of adverse reactions (Holsboer and Ising 2008), considerable reductions in anxiety-related symptoms and sleep disturbances, improved mood, drive, and cognitive symptoms, and reduced suicidality were observed in patients with major depression after therapy with R121919 (a CRF_1_ antagonist). The observed effects were comparable to those exerted by paroxetine (i.e., a selective serotonin reuptake inhibitor) and the affective symptomatology significantly worsened after drug discontinuation (Zobel et al. 2000).

Thus, the available data suggest that blockage of the CRF_1_ receptors may become another strategy for the treatment of depression. However, there is still little information on the potential interactions between the inhibitors of the CRF_1_ receptor and conventional antidepressant therapy. Therefore, we decided to assess the influence of SN003 on the activity of imipramine (a tricyclic antidepressant) and fluoxetine (a selective serotonin reuptake inhibitor) in the FST in corticosterone (CORT)-pretreated rats. SN003, as an inhibitor of the CRF_1_ receptor, completely antagonizes CRF effects without partial or inverse agonist properties, though the observed interaction seems to be at least to some degree non-competitive (Zhang et al. 2003). We found recently that SN003 had a double effect, i.e., apart from its antidepressant potential it also reduces the symptoms of detrusor overactivity (Wrobel et al. 2016). This characteristic of the tested compound is very important from the clinical point of view, since an overactive bladder and depression often co-exist and have a significant impact on the quality of life (Stewart et al. 2003). Similarly to our previous experiments on SN003 (Wrobel et al. 2016), we decided to use the CORT model of depression also in the present study. Literature data suggest that CRF_1_ receptor antagonists may be more effective under stressful conditions when endogenous CRF activity is stimulated (Chaki et al. 2004). We have observed in our previous study that 14-day CORT treatment of rats results in strong elevation of circulating serum CRF levels from 6.41 ± 0.28 pg/ml in the control group to reach 23.39 ± 1.13 pg/ml in the group that had received CORT at a dose of 20 mg/kg/day (Wrobel et al. 2016). Rats treated chronically with glucocorticoids may thus represent a very interesting model to test effects of CRF antagonists.

## Materials and methods

All procedures were performed in accordance with the binding European and Polish law related to experimental studies on animal models, and they were approved by the local ethics committee.

### Animals

All experiments were carried out on naïve female Wistar rats initially weighing 200–225 g. The animals were kept in metabolic cages (3700M071, Tecniplast, USA) in rooms with a natural light/dark cycle, a temperature of 22 ± 1 °C, and a humidity of 60%. They had free access to water and food. The rats were randomly assigned to one of the 10 experimental groups which consisted of 13–15 subjects:

- 1st group (the control group) received saline for 14 days
- 2nd group received CORT (20 mg/kg/day) for 14 days
- 3rd group received CORT (20 mg/kg/day) for 14 days, plus imipramine (30 mg/kg)
- 4th group received CORT (20 mg/kg/day) for 14 days, plus imipramine (15 mg/kg)
- 5th group received CORT (20 mg/kg/day) for 14 days, plus fluoxetine (15 mg/kg)
- 6th group received CORT (20 mg/kg/day) for 14 days, plus fluoxetine (7.5 mg/kg)
- 7th group received CORT (20 mg/kg/day) for 14 days, plus SN003 (1 mg/kg)
- 8th group received CORT (20 mg/kg/day) for 14 days, plus SN003 (0.5 mg/kg)
- 9th group received CORT (20 mg/kg/day) for 14 days, plus imipramine (15 mg/kg), plus SN003 (0.5 mg/kg)
- 10th group received CORT (20 mg/kg/day) for 14 days, plus fluoxetine (7.5 mg/kg), plus SN003 (0.5 mg/kg)

### Drugs

The following drugs were used: corticosterone (CORT; Tocris), imipramine (IMI; Polpharma), fluoxetine (FLX; Eli Lilly), and SN003 (N-(4-Methoxy-2-methylphenyl)-1-[1-(methoxymethyl)propyl]-6-methyl-1H-1,2,3-triazolo[4,5-c]pyridin-4-amine; Tocris). The doses and pretreatment schedules were selected on the basis of our previous experiments (Wrobel et al. 2016). CORT, IMI, and FLX were dissolved in saline, whereas SN003 was dissolved in a minimal volume of about 100 μl of dimethylsulfoxide (DMSO) and diluted in saline, resulting in a final concentration of about 1% DMSO. CORT (20 mg/kg/day) or saline were given subcutaneously (s.c.) for 14 days, IMI (15 or 30 mg/kg) and FLX (7.5 or15 mg/kg) were administered intraperitoneally (i.p.), and SN003 (0.5 or 1 mg/kg) was given intravenously (i.v.). All drugs except for CORT were given as a single dose. The behavioral tests were performed 48 h after the last administration of CORT and 60 min after the injection of IMI, FLX, or SN003. The volume of all administered solutions was 10 ml/kg.

### Forced swim test

The FST was carried out as described before (Porsolt et al. 1977). At first, the animals were placed individually into glass cylinders (height 65 cm, diameter 25 cm) containing 48 cm of water (23–25 °C) and stayed there for 15 min (pretest). After 24 h, rats were retested for 5 min under identical swim conditions. An animal was judged immobile when it was floating passively, performing only slow movements in order to keep its head above the water.

### Locomotor activity

A Digiscan apparatus, an optical animal activity monitoring system (Omnitech Electronics, Inc., Columbus, OH, USA) was used for the assessment of the locomotor activity of rats. The animals were placed individually into activity chambers, and after 15 min of habituation, their horizontal activity was measured automatically for 1 h.

### Measurement of the corticotropin-releasing factor

CRF levels were measured in the hypothalamus, amygdala, and peripheral blood. In order to avoid circadian variations of HPA axis hormones, CORT/saline was given between 8 and 9 a.m. The animals were killed immediately after the behavioral tests. Peripheral blood was collected and the hypothalamus and amygdala were isolated, as described before (Paxinos and Watson 2008). Brain structures were homogenized (Joanny et al. 1989). CRF levels were determined by a high-sensitivity immunoenzymatic test (LBS) according to the instructions of the manufacturer.

### Statistical analysis

The statistical analysis was carried out with GraphPad Prism version 5.01 (GraphPad Software, Inc.). *t* test was used for the comparison of CORT versus saline and one-way analysis of variance (ANOVA) with Dunnett’s or Newman-Keuls Multiple Comparison post hoc test was used for the rest of the statistical comparisons. Dunnett’s post hoc test was applied in order to compare several groups versus the control group, whereas Newman-Keuls Multiple Comparison post hoc test was applied in order to compare several tested groups with each other. All results were presented as the means ± standard error of the mean (SEM). Statistical significance was attained whenever the observed *p* value was less than 0.05.

## Results

### FST

**Fig. 1:**
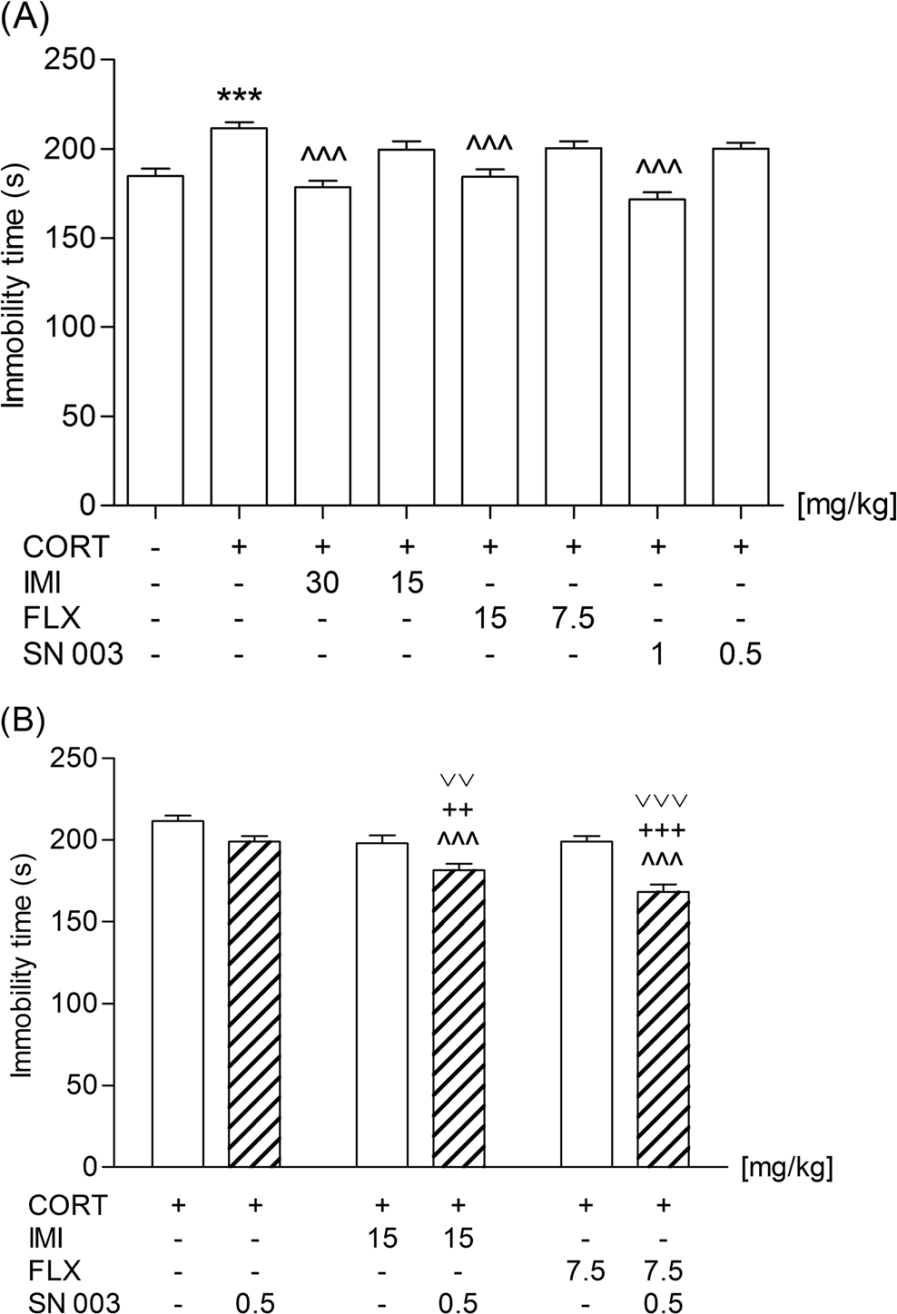
Effect of an acute administration of imipramine (IMI, 15 or 30 mg/kg), fluoxetine (FLX, 7.5 or 15 mg/kg), and SN003(0.5 or 1 mg/kg) on the behavior of rats subjected to 14-day corticosterone treatment (CORT, 20 mg/kg/day) in the forced swim test. The values represent the mean + SEM (*n* = 13–15 animals per group) after a single (**a**) or combined (**b**) injection. ***p < 0.001 versus saline; ^^^*p* < 0.001 versus CORT; ^+++^ *p* < 0.001, ^++^ *p* < 0.01versus CORT plus SN003; ^˅˅˅^ *p* < 0.001, ^˅˅^ *p* < 0.01 versus CORT plus respective antidepressant drug (Dunnett’s or Newman-Keuls Multiple Comparison post hoc test)

As presented in Fig. 1a, 2-week administration of CORT significantly reduced the mobility of rats in the FST (*t*(27) = 4.911; *p* < 0.0001). A single administration of IMI (30 mg/kg; *F*(2,42) = 19.36; *p* < 0.0001), FLX (15 mg/kg; *F*(2,42) = 13.12, *p* < 0.0001), or SN003 (1 mg/kg; *F*(2,40) = 34.70, *p* < 0.0001) reversed the effect induced by CORT. The lower doses of the tested agents (i.e., 15, 7.5, or 0.5 mg/kg, respectively) did not influence the behavior of animals subjected to the repeated CORT treatment. However, co-administration of the sub-active doses of IMI (15 mg/kg) or FLX (7.5 mg/kg) with SN003 (0.5 mg/kg) abolished the pro-depressive activity of the applied glucocorticosteroid (Fig. 1b). One-way ANOVA displayed significant differences between the tested groups: *F*(3,54) = 10.82, *p* < 0.0001 and *F*(3,54) = 24.71, *p* < 0.0001, respectively.

### Locomotor activity

**Fig. 2:**
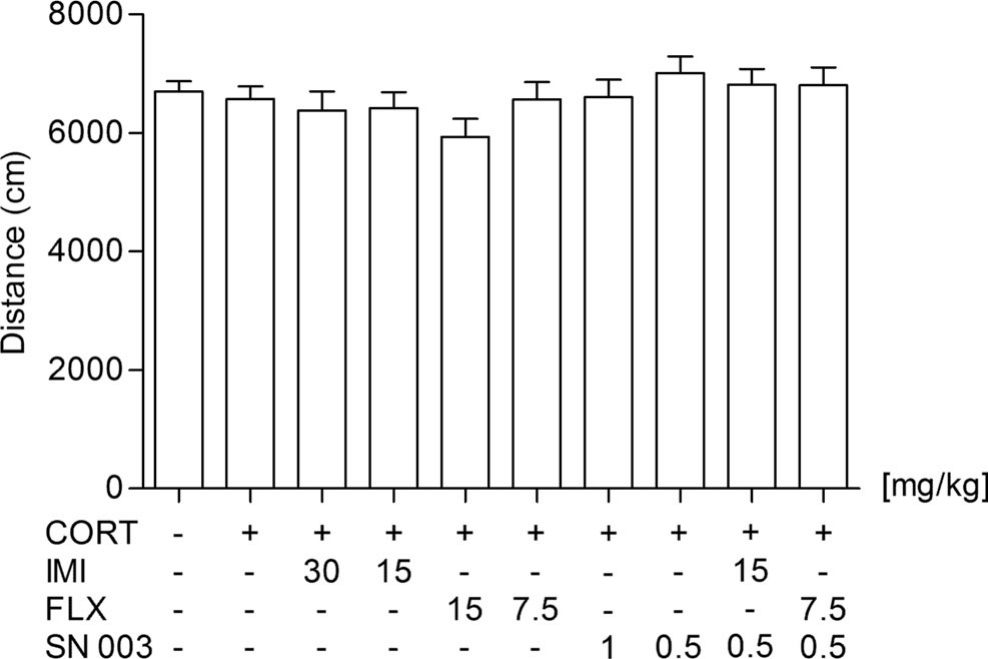
Influence of an acute administration of imipramine (IMI, 15 or 30 mg/kg), fluoxetine (FLX, 7.5 or 15 mg/kg), and SN003 (0.5 or 1 mg/kg) on the locomotor activity of rats subjected to 14-day corticosterone treatment (CORT, 20 mg/kg/day). The values represent the mean + SEM (*n* = 13–15 animals per group)

None of the tested agents injected alone or in combinations affected the locomotor activity of rats as compared to the subjects receiving saline (Fig. 2).

### CRF levels

**Fig. 3:**
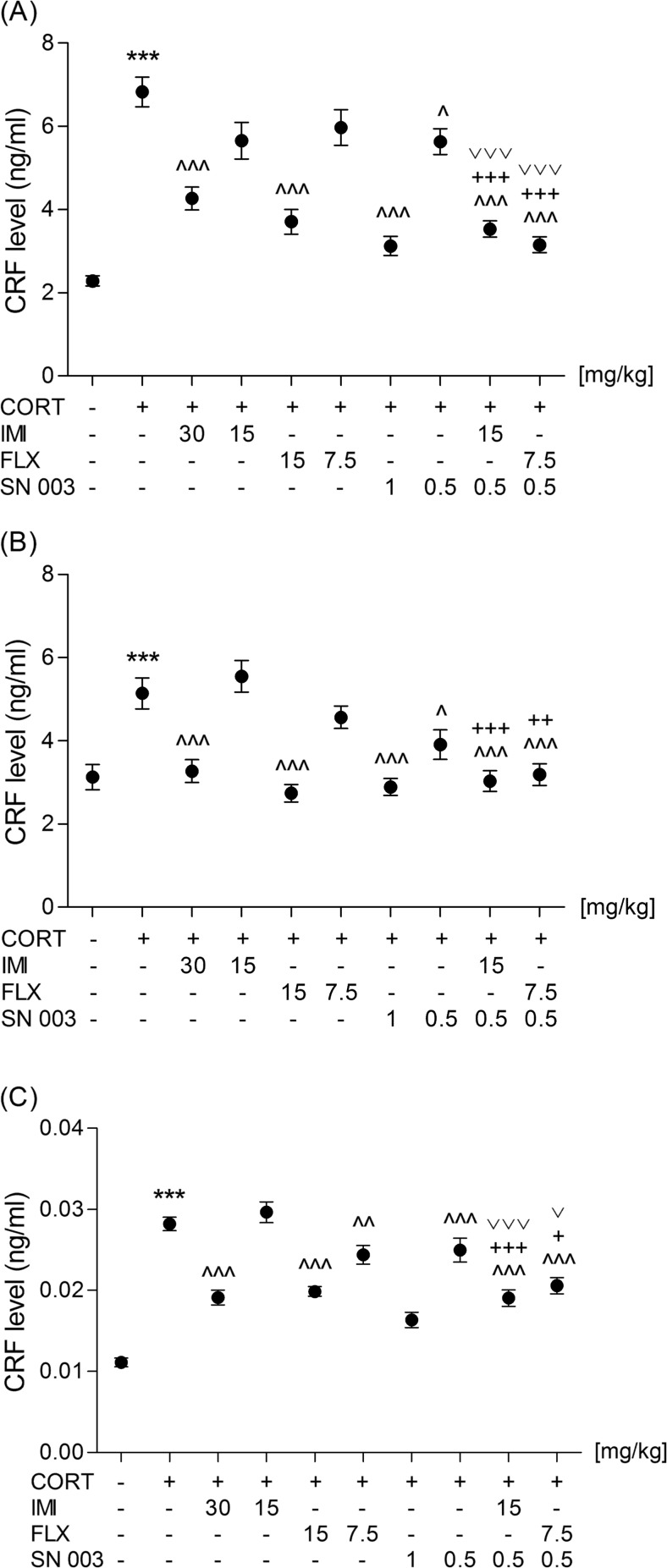
Effect of an acute administration of imipramine (IMI, 15 or 30 mg/kg), fluoxetine (FLX, 7.5 or 15 mg/kg), and SN003(0.5 or 1 mg/kg) given as a single injection or in combination on the CRF levels in hypothalamus (**a**), amygdala (**b**), and peripheral blood (**c**) of rats subjected to 14-day corticosterone treatment (CORT, 20 mg/kg/day). The values represent the mean + SEM (*n* = 13–15 animals per group). ***p < 0.001 versus saline; ^^^*p* < 0.001, ^^*p* < 0.01, ^*p* < 0.05 versus CORT; ^+++^ *p* < 0.001, ^++^ *p* < 0.01, ^+^ *p* < 0.05 versus CORT plus SN003; ^˅˅˅^ *p* < 0.001, ^˅^ *p* < 0.05 versus CORT plus respective antidepressant drug (Dunnett’s or Newman-Keuls Multiple Comparison post hoc test)

After 14-day administration of CORT (20 mg/kg/day), CRF levels were increased in the hypothalamus (*t*(27) = 12.35, *p* < 0.0001), amygdala (*t*(27) = 4.25, *p* < 0.0002), and peripheral blood (*t*(27) = 17.49, *p* < 0.0001), which is shown in Fig. 3. A single administration of IMI, FLX, and SN003 at the higher tested doses reversed this effect in all three tested materials. The lower doses of FLX (7.5 mg/kg) or SN003 (0.5 mg/kg) reduced the elevated CRF levels in the peripheral blood or hypothalamus and amygdala, respectively.

The concurrent administration of a lower dose of the antidepressant drug (IMI or FLX) with the lower tested dose of SN003 significantly abolished the CORT-induced increase in CRF levels in the hypothalamus (*F*(3,54) = 17.68, *p* < 0.0001 and *F*(3,54) = 24.07, *p* < 0.0001), amygdala (*F*(3,54) = 12.01, *p* < 0.0001 and *F*(3,54) = 7.568, *p* = 0.0003), and peripheral blood (*F*(3,54) = 17.78, *p* < 0.0001 and *F*(3,54) = 8.377, *p* = 0.0001).

## Discussion

It has been highlighted previously that the antidepressant-like potential of CRF_1_ receptor antagonists is manifested more profoundly in animals with activated HPA axis, or in models which mimic symptoms of human depression, than in so-called naïve subjects (Yamano et al. 2000; Overstreet et al. 2004; Chaki et al. 2004). In experiments carried out by Yamano et al. (2000), CP-154,526 exerted an antidepressant-like effect in the TST, but only after the administration of interferon-alpha, which is known to induce depression in humans and to increase CRF release from the amygdala and hypothalamus in rats. Overstreet et al. (2004) demonstrated that 14-day administration of this agent significantly increased the swimming time only in the case of rats innately more immobile and genetically prone to depressive-like behavior. Chaki et al. (2004) discovered that an acute administration of another CRF_1_ receptor antagonist (R278995/CRA0450) significantly ameliorated depression-like behavior in various experimental models of depression associated with subchronic stress exposure but it did not show any effect in the FST in rats and the TST in mice. Therefore, in the present study, we decided to use an animal model of depression with experimentally induced elevated CRF level.

As expected, after a 14-day s.c. therapy with CORT (20 mg/kg/day), the rats became less mobile in the FST than the saline-treated control group. This effect was accompanied by a significant increase in CRF levels in the hypothalamus, amygdala, and peripheral blood, which also was not surprising. Lee et al. (2009) suggested that chronic CORT administration increases CRF immunoreactivity in the paraventricular nucleus of the hypothalamus, and this results in the depressive-like behavior of the tested animals. Moreover, it is commonly known that stress-induced hyperreaction of the HPA causes an elevation of hypothalamic and extrahypothalamic CRF levels, as well as raised CRF concentration in the cerebrospinal fluid (CSF) (Arborelius et al. 1999). Both hypothalamic and extrahypothalamic hypersecretion of CRF were observed in depression (Nemeroff et al. 1988).

Our further findings demonstrated that SN003 counteracted CORT-induced prolonged immobility of the tested rats in a dose-dependent manner. Significantly, its antidepressant-like effect was observed after acute treatment, and it was comparable to the effect of conventional antidepressant drugs (IMI and FLX). The outcomes of the present study are generally in line with our previous observations (Wrobel et al. 2016) and the results published by other authors, who focused on the antidepressant potential of the CRF_1_ receptor blockers (Bourke et al. 2014; Chaki et al. 2004; Griebel et al. 2002; Mansbach et al. 1997).

To the best of our knowledge, this is the first report on an interaction between SN003 and conventional antidepressant drugs. A sub-active dose (0.5 mg/kg) of the CRF_1_ receptor antagonist potentiated the antidepressant activity of both IMI (15 mg/kg) and FLX (7.5 mg/kg) in CORT-exposed rats. It should be underlined that the results obtained in the FST were not affected by changes in the locomotor activity of animals, as no significant differences in the overall locomotion between the tested groups were recorded. Though after these preliminary studies we are not able to assess whether the observed interaction is synergistic or only additive, it may open new treatment possibilities in patients suffering from depression.

The available data showed that CRF hypersecretion was normalized after successful antidepressant treatment (De Bellis et al. 1993). Chronic administration of fluoxetine, amitriptyline, desipramine, and mianserine reduced CRF levels in the hypothalamus or CSF (De Bellis et al. 1993; Fadda et al. 1995; Heuser et al. 1998; Veith et al. 1993). In our experiments, an acute dose of the tested agents (SN003, IMI, FLX), given as a single injection or in respective combinations, was sufficient to induce a significant effect. Co-administration of the CRF_1_ receptor blocker with the conventional antidepressants reduced CRF levels in the tested biological material more profoundly than monotherapy, but none of the applied treatments reduced the elevated hypothalamus, amygdala, and peripheral blood CRF to the values recorded for the saline-treated group. Interestingly, SN003 or FLX at the doses that were not potent enough to produce any significant effect in the FST was adequate to partially diminish the elevated CRF concentrations in the hypothalamus and amygdala or peripheral blood, respectively.

In conclusion, our findings provide further evidence that the blockage of CRF action may be useful in the treatment of mood disorders, either as monotherapy or in combination with conventional antidepressant drugs. The concurrent use of well-known antidepressants with CRF_1_ receptor antagonists could be beneficial in terms of safety, since it requires lower doses of the applied agents. However, the results need to be confirmed by additional studies, including experiments with different depression models.
